# A Pruritic Predicament: An Unusual Presentation of Graves’ Disease Complicated by Pregnancy

**DOI:** 10.1155/carm/5816822

**Published:** 2026-08-10

**Authors:** Rob Lawless, Evan Smith, Camryn Slone, Thaddeus Salmon

**Affiliations:** ^1^ University of Kentucky College of Medicine, Lexington, USA, uky.edu; ^2^ Department of Internal Medicine, University of Kentucky College of Medicine, Lexington, USA, uky.edu

## Abstract

Pruritus is a known but uncommon symptom of hyperthyroidism that can be misinterpreted or overlooked in a patient presentation. This is a case report of a 40‐year‐old female who presents with a 3‐month history of severe, worsening pruritus. Further history demonstrated potential signs of hyperthyroidism, and laboratory values demonstrated low TSH levels, high T4 levels, and positive TSH receptor antibodies. A thyroid scan was performed, and increased uptake confirmed the diagnosis of Graves’ disease in this patient. After discovering that the patient was pregnant, treatment with propylthiouracil was begun for the first 12 weeks of her pregnancy, with a switch to methimazole after the first 12 weeks. Graves’ disease must be considered in the differential diagnosis of generalized pruritus, with thorough history and analysis of symptoms essential to determine if underlying hyperthyroidism may be present.

## 1. Introduction

Graves’ disease is an autoimmune condition that primarily affects the thyroid gland and is the most common cause of hyperthyroidism worldwide. Graves’ disease occurs due to thyroid‐stimulating immunoglobulin *G* (IgG), which binds to thyroid‐stimulating hormone (TSH) receptors on the thyroid cell membrane and causes an overproduction of thyroid hormone [1]. This leads to both synthesis of thyroid hormone and growth of the thyroid itself, which can often present as thyromegaly and hyperthyroidism [2]. Patients frequently present with classic symptoms of hyperthyroidism such as tachycardia, systolic hypertension, temperature dysregulation, weight loss, increased appetite, sweating, proximal muscle weakness, warm moist skin, diarrhea, and even hair loss [1, 3]. This case discusses an atypical presentation of Graves’ disease that was subsequently found to be complicated by pregnancy prior to definitive treatment.

Manifestations of Graves’ disease that can narrow the differential diagnosis include ophthalmopathy, which can present with eyelid retraction, increase in retro‐orbital content, and periorbital edema [1]. The diagnosis of Graves’ disease can be made both clinically and using laboratory testing to confirm the diagnosis. Clinical clues for the diagnosis of Graves’ disease include goiter, ophthalmologic signs, and the classic symptoms of hyperthyroidism [4]. If these symptoms are not as clear, or the etiology of symptoms is in question, laboratory testing can also be performed to confirm the diagnosis. Initial testing includes checking TSH, triiodothyronine (T3), and thyroxine (T4) levels. If TSH is low and T3 and/or T4 are elevated, this will confirm the diagnosis of hyperthyroidism [1]. To distinguish Graves’ disease from other causes of hyperthyroidism or destructive thyroiditis, measurement of TSH receptor antibody (TRAb) can be performed. Measurement of TRAb with third‐generation assay has sensitivity and specificity of 97% and 99%, respectively, for the diagnosis of Graves’ disease [1]. Other potential diagnostic measures include thyroid isotope uptake and thyroid ultrasound [4].

Treatment of Graves’ disease is variable, depending on the presentation and severity of symptoms. Most commonly, treatment revolves around stabilizing thyroid hormone production and controlling symptoms that are associated with the condition. Antithyroid drugs such as methimazole and propylthiouracil are commonly used, with methimazole being the preferred agent in pregnant patients after the first trimester [1]. In nonpregnant patients, radioactive iodine therapy is also commonly used, and in severe cases, thyroidectomy can be considered [1]. Care should also be taken in patients who could be or are currently pregnant with Graves’ disease. Fetal complications associated with maternal Graves’ disease include, but are not limited to, fetal hyperthyroidism or hypothyroidism, premature birth, preeclampsia, and fetal goiter [5]. Close fetal and neonatal monitoring is very important to prevent thyroid abnormalities upon birth. This case demonstrated an abnormal presentation of Graves’ disease complicated by pregnancy prior to definitive therapeutic treatment of the condition.

## 2. Case Presentation

A 40‐year‐old woman with a past medical history of chronic lower back pain and abnormal liver function tests (LFTs) presented with a 3‐month history of an urticarial rash with severe pruritus. The pruritus gradually worsened over the course of 3 months and was severe enough at the time of presentation that the patient could no longer sleep. The patient reported that the rash was most predominant on her face and abdomen, and upon examination, it appeared erythematous and urticarial in nature. Although the rash was most predominant in those areas, she states that her pruritus was diffuse throughout her body. She also reported that hot showers make the pruritus worse. She had been taking diphenhydramine in an attempt to relieve her symptoms, but it was providing her no benefit. Although she had been taking amoxicillin from her original country of Haiti, she stated that the rash began before beginning the course of amoxicillin; thus, an allergic medication reaction was removed from the differential. Other symptoms reported by the patient include weakness of her legs, fatigue, tachycardia, weight loss, hair loss, and shortness of breath.

**FIGURE 1:**
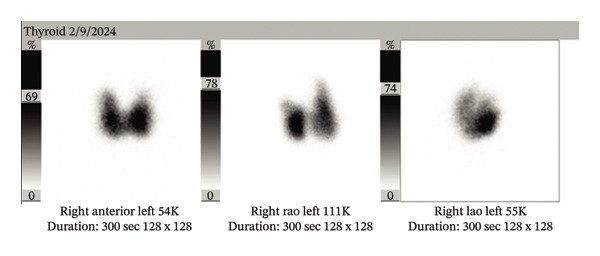
Nuclear medicine thyroid scan showing increased uptake, indicating Graves’ disease.

A comprehensive metabolic panel was ordered, as well as a complete blood count (CBC) with differential and TSH and free thyroxine (FT4) levels. The results showed pertinent findings of TSH < 0.01 mIU/L and FT4 elevated at 3.8 ng/dL. LFTs and alkaline phosphatase and total bilirubin levels were within normal limits. Eosinophil percentage was also found to be normal with a value of 1.0%. TRAb also came back elevated on laboratory testing. A nuclear medicine thyroid scan was then performed which demonstrated diffusely increased thyroid uptake (Figure 1), which confirmed the diagnosis of Graves’ disease. Treatment options were discussed with the patient, which included radioactive iodine‐131 (I‐131) treatment, methimazole, or surgery. The patient opted for permanent I‐131 treatment and acknowledged the necessity for beginning levothyroxine treatment for life after I‐131 treatment. In preparation for the I‐131 treatment, the patient had a serum beta‐human chorionic gonadotropin (beta‐hCG) test performed to ensure that she was not pregnant before beginning treatment. When the beta‐hCG test came back elevated, consistent with a fetus at 8 weeks’ gestation, she was informed that she would not be able to receive I‐131 treatment and was subsequently placed on propylthiouracil 50 mg three times daily for the first 12 weeks of the pregnancy. During the first trimester, the patient was subsequently increased to 100 mg twice daily due to persistently low TSH. After the first trimester, the patient was then switched to methimazole 7.5 mg daily for the remainder of her pregnancy. Propylthiouracil was selected during the first semester due to the risk of methimazole‐induced aplasia cutis in the infant in the first trimester of pregnancy.

At a follow‐up appointment 2 months later to assess her thyroid levels and assessment of her pregnancy, she reported a marked improvement in her symptoms, including her pruritus and other symptoms associated with her hyperthyroidism. Laboratory values for T4 were all within normal limits with a value of 1.3 ng/dL, and T3 was slightly elevated at a value of 197 ng/dL. The patient’s TSG value was found to be 1.23 uIU/mL, also within normal limits. The patient was routinely seen at follow‐up appointments with her endocrinologist and obstetrics and gynecology (OB‐GYN) for monitoring of her condition as well as monitoring complications associated with her pregnancy.

## 3. Discussion

This case demonstrates an abnormal presentation of Graves’ disease in the setting of severe pruritus. Although uncommon, pruritus is a recognized symptom of autoimmune thyroid conditions with an unclear pathophysiology, although previous reports demonstrate these findings in patients with Graves’ disease and elevated serum bile acids [6]. In this patient, the classic finding of ophthalmopathy was not present, but further history provided by the patient showed signs of potential hyperthyroidism, including, but not limited to, tachycardia, temperature dysregulation, weight loss, and appetite loss. This patient’s intolerance to hot showers is notable, as heat intolerance and sweating is a well‐recognized feature of hyperthyroidism. Diminished capacity to regulate body temperature coupled with hyperthyroidism’s postulated direct effects on sweat glands and vascular permeability potentially exacerbated this patient’s pruritus. Another potential etiology of the patient’s pruritus could be bile dysregulation in the setting of her pregnancy. This diagnosis, however, is less likely given that the patient endorsed no change in bowel habits during this time and had normal bilirubin and alkaline phosphatase levels on laboratory investigation. This etiology, however, remained on the differential prior to her diagnosis of Graves’ disease.

Laboratory results, including low TSH, elevated T4, and positive TRAb, were instrumental for diagnosis. These findings were further corroborated by increased uptake on the nuclear scan, which is classic for this condition. The primary treatment, I‐131, was contraindicated due to pregnancy, and propylthiouracil was astutely chosen for the first trimester, followed by methimazole.

The prompt recognition and treatment of this Grave’s disease presentation resulted in significant improvement of both her pruritus and hyperthyroid symptoms. This case emphasizes the need for comprehensive history and detailed analysis, especially in atypical presentations to guide diagnostic approaches. Prompt diagnosis in this case was crucial, potentially preventing preeclampsia, preterm birth, and fetal thyroid dysfunction, all of which are common in pregnant women with undiagnosed hyperthyroidism.

## Funding

No funding was received for this manuscript.

## Consent

No written consent has been obtained from the patient as there are no patient identifiable data included in this case report.

## Conflicts of Interest

The authors declare no conflicts of interest.

## Data Availability

The data that support the findings of this study are available from the corresponding author upon reasonable request.
